# Evaluation of the Probiotic Properties of *Lacticaseibacillus casei* 431^®^ Isolated from Food for Special Medical Purposes^§^

**DOI:** 10.17113/ftb.61.04.23.8045

**Published:** 2023-12

**Authors:** Andreja Leboš Pavunc, Lenkica Penava, Nina Čuljak, Martina Banić, Jasna Novak, Katarina Butorac, Marijana Ceilinger, Jelena Miličević, Danijela Čukelj, Jagoda Šušković, Blaženka Kos

**Affiliations:** 1 University of Zagreb, Faculty of Food Technology and Biotechnology, Laboratory for Antibiotic, Enzyme, Probiotic and Starter Culture Technologies, Pierottijeva 6, 10000 Zagreb, Croatia; 2 Belupo, Pharmaceuticals & Cosmetics Inc., Nutraceuticals, Business Development and Registration, I. Savica 36, 10000 Zagreb, Croatia; 3 Belupo, Pharmaceuticals & Cosmetics Inc., Nutraceuticals, Business Development and Registration, Danica 5, 48000 Koprivnica, Croatia

**Keywords:** *Lacticaseibacillus paracasei* ssp. *paracasei*, food for special medical purposes, functional food, probiotics

## Abstract

**Research background:**

Increasing awareness of the importance of nutrition in health promotion and disease prevention has driven to the development of foods for special medical purposes (FSMPs). In this study, the probiotic strain *Lacticaseibacillus paracasei* ssp. *paracasei* (*Lacticaseibacillus casei* 431^®^) was incorporated into FSMPs to develop an innovative product. The aim was to investigate the influence of the FSMP matrix on the specific probiotic properties of *L. casei* 431^®^ *in vitro*.

**Experimental approach:**

A series of *in vitro* experiments were performed as part of the probiotic approach. After evaluation of antibiotic susceptibility profiles, functional properties such as survival under simulated gastrointestinal tract (GIT) conditions, bile salt deconjugation activities, cholesterol assimilation, antagonistic activity against spoilage bacteria and adhesion to Caco-2 cell line monolayers and extracellular matrix proteins were investigated.

**Results and conclusions:**

The *L. casei* 431^®^ strain, both the lyophilised strain and the strain isolated from the FSMP matrix, effectively survived the simulated adverse gastrointestinal conditions without significant effects of the food matrix. The effect of the FSMP matrix on the deconjugation activity of the bile salts of *L. casei* 431^®^ was minimal; however, cholesterol assimilation was increased by 16.4 %. *L. casei* 431^®^ had antibacterial activity against related lactic acid bacteria regardless of whether it was used in FSMPs or not. Conversely, the probiotic strain isolated from FSMP matrix had significantly higher inhibitory activity against six potential pathogens than the lyophilised culture. The autoaggregation ability of the *L. casei* 431^®^ cells was not affected by the FSMP matrix. The adhesion of *L. casei* 431^®^ bacterial cells to the extracellular matrix proteins was reduced after treatment with proteinase K, with the highest adhesion observed to laminin. The adhesion of *L. casei* 431^®^ reduced the binding of *E. coli* 3014 by 1.81 log units and the binding of *S*. Typhimurium FP1 to Caco-2 cell lines by 1.85 log units, suggesting the potential for competitive exclusion of these pathogens.

**Novelty and scientific contribution:**

The results support the positive effect of the FSMP matrix on the specific probiotic properties of *L. casei* 431^®^, such as antibacterial activity, bile salt deconjugation and cholesterol assimilation, while the incorporation of this probiotic strain adds functional value to the FSMPs. The synergistic effect achieved by the joint application of *L. casei* 431^®^ and innovative FSMP matrix contributed to the development of the novel formulation of an improved functional food product with added value.

## INTRODUCTION

Nowadays, global trends are placing increasing importance on functional nutrition as a basis for human well-being and health management. Increasing consumer awareness of the relationship between nutrition and health and the production of state-of-the-art food with positive health effects or probiotic food supplements opens up opportunities for both consumers and the food and pharmaceutical industries. Likewise, nutrition science points out the importance of nutrition in disease prevention, which, along with the avoidance of harmful modern lifestyle habits, can reduce the risks of metabolic syndrome that consists of a group of risk factors: elevated blood pressure or blood sugar concentration, elevated cholesterol levels and increased abdominal fat (*1*). Metabolic syndrome is associated with increased incidence of cardiovascular disease, diabetes, obesity and cancer. This has stimulated the intensification of global research activities in the field of nutrigenomics, the development of personalised foods, and foods intended to prevent the occurrence or alleviate the consequences of acute or chronic diseases. Food for special medical purposes (FSMPs) is intended for the complete or partial nutrition of patients with a disturbed ability to metabolise food or for patients who have special medical requirements (*2*).

Another aspect of nutrition as a health-promoting concept is functional food based on the addition of probiotics that promote cognitive response, improve immune system, and overall well-being (*3*). The molecular mechanisms responsible for probiotic effects can be associated with a range of microbial metabolites and molecules exposed at the cell surface, such as surface (S)-layer proteins, exopolysaccharides, bacteriocins, which are also mediated by enzymatic activities of bioactive peptides produced by probiotic proteolytic enzymes (*4*-*6*). According to the European legislation, probiotic preparations are categorised as functional food ingredients or as food supplements (*7*). According to the US Food and Drug Administration, probiotics are not only used as health-promoting food supplements (nutraceuticals), but also as live biotherapeutic products (LBPs) (*8*). The quality assurance of probiotics as LBPs on the European market is established by the European Pharmacopoeia Commission (*9*). Probiotics are used to improve digestive health and strenghten the immune system. The International Scientific Association for Probiotics and Prebiotics (ISAPP) has supported and reaffirmed the FAO/WHO definition of a probiotic as live microorganisms that, when administered in adequate amounts, confer a health benefit on the host (*10*).

The most commonly recognised probiotic strains come from the genera of lactic acid bacteria (LAB) that are generally recognized as safe (GRAS). Traditionally associated with fermented food, LAB favourably influence the intestinal microbiota balance (*11*, *12*). Many of them are distributed in various probiotic products such as functional foods, infant formulations, foods for special dietary and medical purposes, as well as dietary supplements (nutraceuticals) in the form of capsules, tablets, powders or solutions in lyophilised and microencapsulated form (*13*-*15*). *Lacticaseibacillus paracasei* strains have been documented in numerous studies as bacteria with strong probiotic activity (*16*). These are Gram-positive, non-sporogenic, facultatively heterofermentative bacteria that are indigenous to the intestinal microbiota. The beneficial effects of *Lacticaseibacillus paracasei* strains have been demonstrated in patients with various digestive disorders, infectious diseases, obesity and depression in clinical studies (*17*).

Various methods for determining the content, purity and identity of microorganisms in probiotic products have been described in the scientific literature, but the influence of the product matrix on the functionality of the probiotic culture is not widely known (*18*).

Therefore, the aim of this work is to develop novel FSMP product with the addition of the probiotic *Lacticaseibacillus paracasei* (*Lacticaseibacillus casei* 431^®^) strain. This new product would be intended for malnourished patients when, due to medical reasons, it is not possible to utilize the nutritional needs from a normal diet. Probiotic strains in the product may also provide additional functional value to this final preparation.

The impact of the food matrix on the functional properties of *L. casei* 431^®^ was tested through a series of *in vitro* experiments as part of the proposed probiotic concept, which include sensitivity or resistance to antibiotics according to EFSA (*19*), survival under simulated gastrointestinal tract (GIT) conditions, deconjugation activity of bile salts, cholesterol assimilation, antagonistic capacity including potential bacteriocin activity and ability of adhesion on the Caco-2 cell line monolayer and extracellular matrix proteins.

## MATERIALS AND METHODS

### Food for special medical purposes with Lacticaseibacillus paracasei ssp. paracasei strain (L. casei 431^®^)

Probiotic culture *Lacticaseibacillus casei* 431^®^ (Chr. Hansen Holding A/S, Hørsholm, Denmark) was tested as a lyophilised pure culture and after isolation from the samples of FSMPs. The lyophilised pure culture contains freeze-dried cells of *Lacticaseibacillus paracasei* sp. *paracasei*, as well as sucrose, maltodextrin and sodium ascorbate.

The food matrix of FSMPs was produced in the development laboratories of Belupo d.d. (Koprivnica, Croatia) and in the pilot plant of Podravka d.d. (Koprivnica, Croatia). FSMPs contain maltodextrin, sunflower oil, milk proteins, sucrose, dietary fibre (inulin), medium-chain triglycerides from coconut oil, minerals (calcium lactate, sodium chloride, iron lactate, zinc sulfate, manganese sulfate, copper sulfate, chromium(III) chloride, potassium iodide, sodium molybdate, sodium selenate, sodium fluoride, sodium selenite, copper gluconate), vitamins (l-ascorbic acid, dl-alpha tocopheryl acetate, nicotinamide, calcium d-pantothenate, retinyl acetate, cholecalciferol, pyridoxine hydrochloride, thiamine hydrochloride, phytomenadione, riboflavin, folic acid, d-biotin, cyanocobalamin, sodium l-ascorbate), emulsifier (soy lecithin), glucose syrup powder, anti-caking agent (silicon dioxide), acidity regulators (citric acid, dipotassium phosphate, dimagnesium phosphate, disodium phosphate, magnesium hydrogen carbonate), sweetener (acesulfame K), thickeners (xanthan gum, carrageenan), antioxidant (sodium ascorbate), aroma, choline bitartrate and mass fraction of *L. paracasei* culture (Chr. Hansen Holding A/S) 0.25 % (*N*=2.0·10^7^ CFU/g).

The survival of the probiotic strain in FSMP matrix during 24 months of storage at room temperature was determined by the agar plate counting method using de Man, Rogosa, Sharpe (MRS) agar (Merck, Darmstadt, Germany). Plates were incubated anaerobically at 37 °C for 48 h.

### Sequencing of the 16S rRNA gene

The taxonomic identification of the strain after recovery from FSMPs was confirmed by sequencing the 16S rRNA gene (Macrogen, Amsterdam, Netherlands) using an automatic 96-capillary device ABI 3730xl Genetic Analyzer (Applied Biosystems, Waltham, MA, USA), which operates on the principle of the Sanger dideoxy method, in which DNA synthesis is stopped by the incorporation of 2',3'-dideoxynucleotides (ddNTPs). The obtained results were compared with known sequences in the National Center for Biotechnology Information (NCBI) database using the Nucleotide Basic Local Alignment Search Tool (BLASTn) algorithm available at http://www.ncbi.nlm.nih.gov/blast/ (*20*).

### Survival of the strain in simulated GIT juices

The cumulative effect of simulated GIT juices on the survival of strain *L. casei* 431^®^ was investigated (*21*). *L. casei* 431^®^ cells and FSMP matrix were exposed to simulated gastric juice for 2 h, then centrifuged to remove the gastric juice and then the cells or the matrix were subjected to a 4-hour incubation in simulated small intestinal juice. Changes in the total viable count were monitored periodically every hour during the treatment.

### Quantitative determination of bile salt hydrolase activity and cholesterol assimilation

Bile salt hydrolase (BSH) activity was determined by quantifying the cholic acid released from the conjugated bile salt sodium taurocholate (Difco, Detroit, MI, USA) by the solvent extraction method, whereas the ability of *L. casei* 431^®^ to assimilate cholesterol (AppliChem, Darmstadt, Germany) added to the MRS broth (0.2 mg/mL) with 3 mg/mL oxgall (Difco) was evaluated by the modified *o*-phthalaldehyde method in the supernatant, cell extract and in the washing buffer where precipitated cholesterol from the supernatant was redissolved (*14*).

### Antibiotic susceptibility testing

Susceptibility to antibiotics was examined by E-test strips (M.I.C. Evaluator^TM^, Oxoid, Ltd, Basingstoke, UK) according to Leboš Pavunc *et al*. (*22*) and the obtained results were interpreted according to EFSA (*19*).

### Antimicrobial activity

Antimicrobial activity was tested with an agar-well diffusion assay, an agar spot test (*13*) and with a microplate growth inhibition assay of cell-free supernatants of *L. casei* 431^®^ (*15*). Samples for agar spot test were 10-fold dilutions of lyophilised pure cultures of *L. casei* 431^®^ and *L. casei* 431^®^ from FSMP matrix that were inoculated on the surface of the MRS agar plates. Samples for the agar-well diffusion assay and microplate growth inhibition assay of cell-free supernatants of *L. casei* 431^®^ were obtained after cultivation of a lyophilised pure culture of the *L. casei* 431^®^ strain and from the FSMP matrix in the MRS broth. The antagonistic activity of the *L. casei* 431^®^ strain evaluated by the agar-well diffusion assay was expressed as the diameter of the inhibition zones.

### Aggregation assays

Auto- and coaggregation assays during 5 h of incubation in phosphate-buffered saline (PBS; pH=7.4; Gibco, Paisley, Scotland, UK) were performed using a microplate reader INFINITE^®^ 200 PRO (Tecan, Männedorf, Switzerland) (*21*, *23*). The autoaggregation and coaggregation were monitored for 5 h in PBS (pH=7.4) and the obtained results were expressed as a percentage of aggregated cells.

### Adhesion on Caco-2 cells and extracellular matrix proteins

Adhesion properties of *L. casei* 431^®^ were tested according to Novak *et al.* (*11*). Caco-2 cells were first seeded on a 96-well microtiter plate in a final volume of 100 μL Dulbecco's modified Eagle’s medium/Ham's F-12 medium (DMEM/F12; Capricorn Scientific GmbH, Ebsdorfergrund, Germany) enriched with 10 % foetal bovine serum (Thermo Fisher Scientific, Rochester, NY, USA) and l-glutamine (Sigma-Aldrich, Merck, Schnelldorf, Germany). After that, Caco-2 cells were inoculated with a bacterial suspension at the multiplicity of infection (MOI) 50, 10 or 2 and then stained in a suspension with Incucyte® Rapid Red dye (Sartorius, Schloß Holte-Stokenbrock, Germany). After fixation and washing with PBS, the Caco-2 cells were stained with 4',6-diamidino-2-phenylindole (DAPI dihydrochloride; Thermo Fisher Scientific, Waltham, MA, USA) and the adherent *L. casei* 431^®^ were detected on the EVOS FLc Cell Imager device (Thermo Fisher Scientific, Waltham).

Binding of *L. casei* 431^®^ to human extracellular matrix proteins (fibronectin, laminin and type I collagen), with and without the treatment with proteinase K (Invitrogen™, Waltham, MA, USA) was also examined (*14*).

### Competitive exclusion assay on the Caco-2 cell line

To assess the ability of the *L. casei* 431^®^ strain to exclude enteropathogens *Salmonella enterica* Typhimurium FP1 and *Escherichia coli* 3014, exclusion and adhesion assay was performed on the Caco-2 cell monolayers (*24*, *25*). Viable adhered *L. casei* 431^®^, *E. coli* and *S.* Typhimurium (CFU/mL) were determined by the spot-plate method on MRS, Rapid (Bio-Rad, Dubai, United Arab Emirates) and XLD (Biolife, Milan, Italy) agar plates, respectively (*24*, *25*).

### Statistical analyses

The results were expressed as mean values of three independent trials±standard deviation. Statistical significance was determined using one-way analysis of variance. Pairwise differences between group means were determined using Tukey’s HSD test for pairwise comparisons after the analysis of variance (Statistics Kingdom) (*26*). Statistical differences between treatments were considered significant at *p*<0.05.

## RESULTS AND DISCUSSION

As a first criterion for achieving a positive effect on health, probiotic preparations should contain live cells of the probiotic strain in amounts higher than 10^6^ CFU/g of the product (*27*). Accordingly, an important technological parameter is monitoring the survival of probiotic bacteria during the preparation and storage of the product within the declared expiration date. Therefore, the survival of *L. casei* 431^®^ in the product was tested during 24 months of storage at room temperature. The initial number of viable cells of *L. casei* 431^®^ in FSMP matrix was 2.0·10^7^ CFU/g, which did not change remarkably after 24 months of storage at room temperature and was above the required number of live probiotic cells. To validate the probiotic culture added to FSMPs, phenotypic and genotypic characterisation of *L. casei* 431^®^ was undertaken. *L. casei* 431^®^ was recovered from a sample of FSMPs on MRS agar. After culturing 20 single colonies on MRS agar overnight, the isolated bacteria were identified and confirmed as *L. casei* 431^®^ by 16S rRNA gene sequencing.

In order to investigate the influence of the food matrix on the *in vitro* functionality of *L. casei* 431^®^, a series of experiments were conducted to compare the specific probiotic properties of the lyophilised culture and the isolate from the FSMP matrix. According to the EFSA Scientific Opinion (*28*), bacterial strains added to food must be sensitive to the relevant spectrum of antibiotics of human and veterinary importance. According to EFSA (*19*), minimum inhibitory concentrations (MICs) of 4, 1, 32, 64, 4, 4, 64 and 4 μg/mL are proposed for ampicillin, erythromycin, gentamicin, kanamycin, clindamycin, chloramphenicol, streptomycin and tetracycline, respectively for *Lacticaseibacillus paracasei*. According to the results, *L. casei* 431^®^ is susceptible to all tested antibiotics, with certain antibiotics inhibiting the growth even at concetrations below the proposed cut-offs (0.19 for ampicillin, 32 for streptomycin, 0.94 for erythromycin, 0.047 for clindamycin and 0.25 μg/mL for tetracycline). There are no differences in the antibiotic susceptibility profiles (MIC values) between the lyophilised isolate and the isolate from the FSMP matrix (data not shown).

**Fig. 1:**
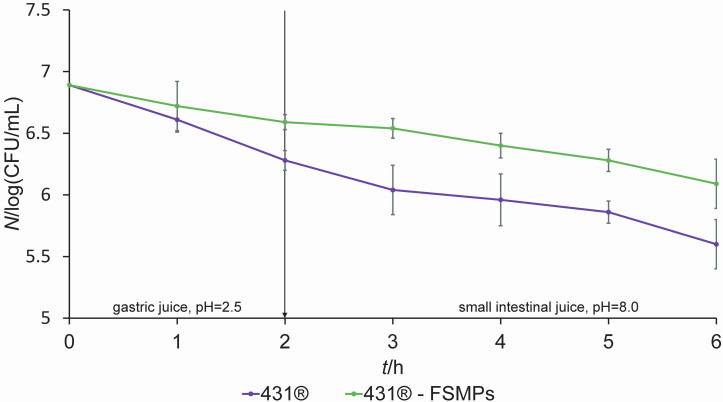
The cumulative effect of simulated gastric juice (pH=2.0, *t*(incubation)=2 h) and simulated small intestine juice (pH=8.0, *t*(incubation)=4 h) on the survival of *Lacticaseibacillus casei* 431^®^ as lyophilised pure culture and isolates from food for special medical purposes (FSMPs). The results are expressed as mean values of three independent experiments, and error bars represent standard deviation. There were no statistically significant differences among the compared results (p<0.05)

The potential of *L. casei* 431^®^, both the lyophilised culture and the cultures grown from the isolates from the FSMP matrix, to survive under simulated GIT conditions was tested, as well as the effect of food matrices on its survival under these conditions. Survival of the tested strains was monitored at one-hour intervals for 2 h in simulated gastric juice and after direct transit to simulated small intestinal juice during a 4-hour incubation (Fig. 1).

Both cultures successfully survived the adverse conditions. The survival rate of the lyophilised pure culture compared to the isolate from the FSMP matrix differed by only about half a log unit. There were no significant differences in the survival among lyophilised cultures and isolate from the FSMPs when *L. casei* 431^®^ cells were exposed to simultaneous stress conditions of the GIT. González-Vázquez *et al.* (*29*) also exposed two *Lacticaseibacillus casei* strains, Shirota and J57, to unfavourable GIT conditions. The viability of *L. casei* J57 did not change remarkably during a 4-hour exposure to simulated gastric juice conditions, while the survival rate of the strain *L. casei* Shirota dropped drastically after only 1 h of exposure (below 40 %) and no living cells were present after 2 h. The strains were also exposed for 4 h to different bile salts. Both strains showed the highest hydrolase activity towards taurocholic acid and moderate activity towards oxgall bile salts compared to the others, and their survival rate under adverse GIT conditions was at least 50 %.

Since oxgall is produced from a large amount of fresh bile by rapid evaporation of water and has a complex composition with a negative synergistic effect of specific compounds on bacterial survival, the very high viable cell counts of *L. casei* 431^®^, observed even after 4 h of exposure to simulated conditions of the small intestine, are a promising result (Fig. 1). The high survival rate under simulated gastric juice conditions might also be associated with cross-resistance mechanisms involving modifications of the peptidoglycan structure in both the core peptide and/or the sugar moieties. Adaptation of LAB to acid stress involves a mechanism to maintain cytoplasmic pH, and many of these systems also provide cross-protection when cells are exposed to other stressors such as high concentrations of bile salts (*30*, *31*).

A high concentration of bile salts is the main obstacle to the colonization of probiotic strains in the *ileum terminalis* since bile salts express antimicrobial activity against Gram-positive microorganisms. One of the mechanisms of resistance to bile salts is established through the activity of BSH through deconjugation of bile salts (*32*). Therefore, the growth of *L. casei* 431^®^, as a lyophilised pure culture and isolate from the FSMPs matrix, in the presence of bile salts and the deconjugation activity of the cells towards bile salts were tested using a qualitative method (data not shown). The sodium salt of taurocholic acid was used as it is the most abundant bile salt in human bile. An increase in BSH levels is also associated with a greater ability to reduce the concentration of blood cholesterol, which is considered a desirable function of probiotics (*32*). Therefore, spectrophotometric determination of precipitated, assimilated and dissolved cholesterol was carried out after 24 h of cultivation of *L. casei* 431^®^ as a lyophilised pure culture and as an isolate from the FSMPs in the presence of bile salts (data not shown). Analysis of the growth of *L. casei* 431^®^ strains in the MRS medium containing 2 mg/mL sodium taurocholate revealed that deconjugation activity was evident in both the lyophilised pure culture and the isolate from FSMPs ((13.00±0.04) % and (14.5±0.6) %, respectively). Although the deconjugation activity of the strain isolated from the FSMP matrix was slightly higher, we were not able to determine the influence of the food matrix on this activity and the ability of the strain to grow (absorbance at *λ*=620 nm was 0.38±0.04 for the pure lyophilised cultre and 0.42±0.05 for the isolate from the FSMPs) in the presence of bile salts. After 24 h of growth in the presence of cholesterol and bile salts, the cells of *L. casei* 431^®^ from a lyophilised pure culture removed 54.77 % of the cholesterol from the MRS medium and assimilated 32.85 % of the cholesterol. In contrast, *L. casei* 431^®^ cells isolated from the FSMP matrix assimilated 49.25 % of the cholesterol, while 45.85 % of the cholesterol was deposited in the solution during cell growth. These cells removed 96.52 % of the cholesterol from the MRS medium. Since *L. casei* 431^®^ cells added to the FSMP matrix assimilated 49.25 % of the cholesterol compared to 32.85 % of the lyophilised pure culture, it is reasonable to assume that a food matrix contributed to increased cholesterol assimilation by 16.4 %. Lactobacilli are thought to remove cholesterol due to deconjugation of bile salts by the BSH (*32*). Deconjugation of bile salts leads to a drop in pH value, which destabilises cholesterol micelles, resulting in precipitation of cholesterol by free bile acids. Bacterial cells remove cholesterol from the medium by adsorption or absorption by incorporating cholesterol into the cell membrane (*33*). According to the recent literature data, the administration of functional food containing probiotic bacteria was effective in cholesterol assimilation under simulated GIT conditions (*34*). Nutrient-rich food matrices such as FSMPs might be associated with a higher capacity of *L. casei* 431^®^ from the FSMP matrix to assimilate cholesterol compared to MRS medium. In this context, Chaiyasut *et al.* (*35*) reported that the *L. paracasei* HII01 strain significantly reduced the level of total cholesterol, triglycerides and tumour necrosis factor and lipopolysaccharide in patients with hypercholesterolaemia while increasing levels of high-density lipoprotein (HDL) cholesterol, total antioxidant capacity, and propionic and lactic acids. Thus, the addition of the strain *L. paracasei* HII01 improved the blood lipid profile and reduced oxidative stress (*35*). The ability of certain probiotic strains to decrease cholesterol concentrations may have an impact on the prevention or incidence of hypercholesterolaemia, which is one of the major causes of cardiovascular disease (*36*). Albano *et al.* (*37*) showed that among 58 LAB, the strains *Lacticaseibacillus paracasei* ssp. *paracasei* SE160 and VC213 reduced cholesterol concentration in the medium by 55 and 45 %, respectively, and were subsequently tested for their ability to decrease cholesterol amounts in cheese production. All strains were present in cheese in counts higher than 10^7^ CFU/g and the highest reduction in cholesterol amounts in cheese (up to 23 %) was achieved with *L. paracasei* ssp. *paracasei* VC213. Overall, the implementation of probiotic lactobacilli with cholesterol-lowering ability contributes to the development of novel functional foods, FSMPs and other nutraceuticals or fermented dairy products with reduced cholesterol content and important functional properties. The application of these strains extends to the development of nutritional supplement formulations intended for the prevention of dyslipidaemia, coronary disease and metabolic syndrome.

**Fig. 2:**
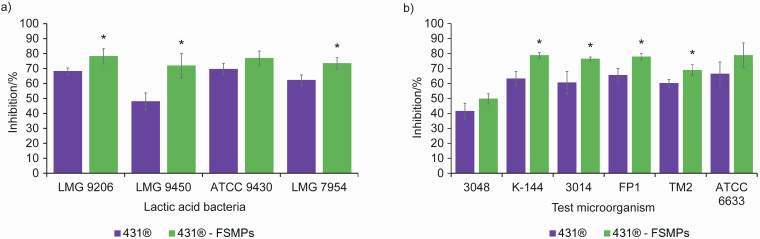
The antibacterial activity of cell-free supernatants of *Lacticaseibacillus casei* 431^®^ as lyophilised pure culture and an isolate from the food for special medical purposes (FSMPs) after 24 h of incubation tested by the microplate growth inhibition assay against: a) lactic acid bacteria strains *L. plantarum* LMG 9206, *Lc. lactis* ssp. *lactis* LMG 9450, *E. faecium* ATCC 9430 and *Ln. mesenteroides* LMG 7954 and b) test microorganisms *S. aureus* 3048, *S. aureus* K-144, *E. coli* 3014, *S. enterica* serovar Typhimurium FP1, *B. cereus* TM2 and *B. subtilis* ATCC 6633. Data show the average of three independent replicates. *Significantly different (p<0.05) from the lyophilised pure culture

As mentioned above, the BSH activity of probiotic lactobacilli can generally enhance antimicrobial activity in the human GIT. When implemented as probiotic-fortified food, it would be optimal if the designed food matrix strengthened the antipathogenic capacity of the applied probiotic strain. Therefore, the antimicrobial capacity of the two different formulations of *L. casei* 431^®^ was evaluated. The antimicrobial activity of *L. casei* 431^®^ cells propagated from a lyophilised pure preparation and of an isolate from the FSMPs against microorganisms that are potential food contaminants and causative agents of various infections was examined using the agar spot test (Table 1) and agar-well diffusion assay (Table 2). The antimicrobial acitivity of the cell-free supernatants was investigated using the microplate growth inhibition assay (Fig. 2).

**Table 1:** Antibacterial activity of *Lacticaseibacillus casei* 431^®^ as lyophilised pure culture and as an isolate from the food for special medical purposes (FSMPs) against lactic acid bacteria as related Gram-positive microorganisms tested by the agar spot test

Lactic acid bacteria	Effective inhibition ratio (EIR) (*d*_i_-*d*_c_)/*d*_c_	
*L. casei* 431^®^	*L. casei* 431^®^ FSMPs
*Lactiplantibacillus plantarum* LMG 9206	(0.5±0.1)^a^	(0.67±0.09)^a^
*Lactococcus lactis* ssp. *lactis* LMG 9450	(0.4±0.1)^a^	(0.6±0.1)^a^
*Enterococcus faecium* ATCC 9430	(0.58±0.03)^a^	(0.62±0.04)^a^
*Leuconostoc mesenteroides* LMG 7954	(0.27±0.05)^b^	(0.53±0.08)^a^

*d*_i_=inhibition diameter, *d*_c_=diameter of the spot with grown colonies, EIR<0.5=weak inhibition, 0.5<EIR>1.5=medium inhibition, EIR>1.5=strong inhibition

The results are expressed as the mean value±standard deviation of three separate experiments. Different letters in superscript indicate statistically significant differences among data in the same column (Tukey’s HSD test, p<0.05, *N*=3)

**Table 2:** Antibacterial activity of *Lacticaseibacillus casei* 431^®^ as lyophilised pure culture and as an isolate from the food for special medical purposes (FSMPs) against potential pathogens as test microorganisms tested by the agar-well diffusion assay and expressed as the diameter of the zone of inhibition

Test microorganism	*d*(inhibition zone)/mm	
*L. casei* 431^®^	*L. casei* 431^®^ FSMP
*Staphylococcus aureus* 3048	(3.11±0.05)^b^	(8.67±0.09)^a^
*Staphylococcus aureus* K-144	(10.01±0.09)^b^	(13.71±1.21)^a^
*Escherichia coli* 3014	(11.2±1.1)^b^	(14.3±1.2)^a^
*Salmonella* Typhimurium FP1	(9.3±1.1)^b^	(14.2±1.5)^a^
*Bacillus cereus* TM2	(12.33±0.09)^b^	(16.67±1.3)^a^
*Bacillus subtilis* ATCC6633	(9.2±1.4)^b^	(13.3±1.8)^a^

The results are expressed as the mean value±standard deviation of three separate experiments. Different letters in superscript indicate statistically significant differences among the data in the same column (Tukey’s HSD test, p<0.05, *N*=3)

According to the results, the antimicrobial activity of *L. casei* 431^®^ was not significantly affected by whether the strain was derived from the FSMPs or not, with the exception of antimicrobial activity against the related bacterium *Leuconostoc mesenteroides* LMG 7954 (Table 1). The isolate from the FSMPs inhibited moderately while the cells from the lyophilised pure culture inhibited related LAB strains only weakly.

When testing antimicrobial activity against common food contaminants, cells from the FSMP matrix had a significantly higher inhibitory effect, suggesting that the matrix enhances the inhibitory effect of *L. casei* 431^®^ probably by increasing the concentration of antimicrobial metabolites produced as a nutritionally rich medium compared to the MRS medium (Table 2).

When testing the antibacterial activity of the supernatants of *L. casei* 431^®^ culture, cells from the FSMP matrix showed a significantly stronger inhibitory effect against the tested microorganisms than the cells recovered from lyophilised culture (Fig. 2). *L. casei* 431^®^ cells from the FSMP matrix generally showed higer inhibition of 12 %, while the inhibition determined with related LAB strains were 10 % higher on average, with the exception of 24 % determined for *Lactococcus lactis* ssp. *lactis* LMG 9450 (Fig. 2). It can be speculated that the antimicrobial activity was achieved primarily through the synthesis of a number of inhibitory metabolites *in situ,* including organic acids, hydrogen peroxide, ethanol, diacetyl, acetaldehyde, as well as bacteriocins that mainly inhibit Gram-positive microorganisms (*38*). Many studies have shown different mechanisms of antimicrobial activity of lactobacilli using *in vitro* tests (*19*, *39*-*41*). Islam *et al.* (*42*) demonstrated the strong antibacterial activity of *Lacticaseibacillus paracasei* ssp. *paracasei-1* isolated from traditional yoghurt. Radulović *et al.* (*43*) showed that three autochthonous traditional cheese isolates of *Lacticaseibacillus paracasei* strongly inhibited *Staphylococcus aureus*, *Escherichia coli* and *Bacillus subtilis*. In general, testing the antimicrobial activity of strains cultured in liquid media is advantageous due to the facilitated diffusion of antimicrobial metabolites such as lactic acid, bacteriocins, *etc.* Given that bacteriocins are primarily active against closely related species, and that the cell-free supernatant of *L. casei* 431^®^ showed strong activity against related LAB, it can be assumed that the antimicrobial activity is also bacteriocin based. This needs to be further investigated, and as a first step, the antimicrobial activity of the neutralised supernatant should be tested to eliminate the inhibitory effect of the lactic acid produced (Fig. 2a).

**Fig. 3:**
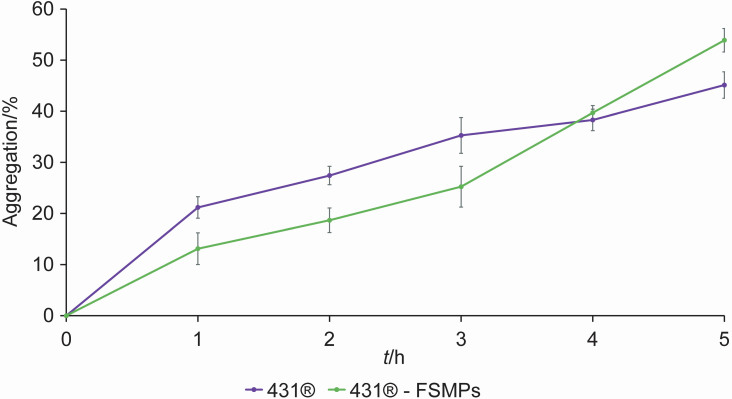
Autoaggregation of *Lacticaseibacillus casei* 431^®^ strains grown from lyophilised pure culture and an isolate from the food for special medical purposes (FSMPs). The results are expressed as mean value of three independent experiments and error bars represent standard deviation. There were no statistically significant differences between the autoaggregation properties of lyophilised culture or *L. casei* 431^®^ isolate from the FSMP (p<0.05)

In addition to antimicrobial activity, adhesion is also reported as an important criterion for probiotic strains. Adhesion to intestinal epithelial cells is an important prerequisite for the colonization with probiotic strains, because it reduces their removal by intestinal peristalsis and gives them a competitive advantage over other bacteria for a longer transit in the GIT ecosystem. Aggregation is a necessary prerequisite for the adhesion of probiotic strains to the intestinal epithelial cells (*7*). It is the ability of bacterial cells to settle during growth, with autoaggregation involving cells of the same bacterial strain, while genetically different cells coaggregate. The autoaggregation of *L. casei* 431^®^ cells, as a pure lyophilised culture and as an isolate from the FSMP matrix, is shown in Fig. 3. Autoaggregation of lyophilised pure *L. casei* 431^®^ culture during the first 3 h of incubation was approx. 10 % higher than the autoaggregation of the *L. casei* 431^®^ isolate from the FSMP matrix, but without statistical significance. In the last, fifth hour of the experiment, the percentage of autoaggregation of the pure culture was lower (45.13 %) than of the isolate from the FSMP matrix, which was 53.90 % (Fig. 3). In lactobacilli, two types of proteins are responsible for aggregation, namely soluble proteins and surface proteins (*25*, *44*). The smallest protein with a size of 2 kDa that mediates autoaggregation was found in *Lactobacillus gasseri* 2459. The protein with a molecular mass of 200 kDa, which is directly involved in aggregation, was found in *Lacticaseibacillus paracasei* ssp. *paracasei* BGSJ2-8 (*45*).

The factors that promote the coaggregation of lactobacilli differ from each other in terms of molecular mass and primary structure. Considering coaggregation, the probiotic bacteria that successfully coaggregate and also exhibit strong antimicrobial activity can create a barrier that prevents the colonization of pathogens. Additionally, coaggregation with other LAB that tolerate low pH values promotes the colonization of the intestine with bifidobacteria. Here we tested the coaggregation of *L. casei* 431^®^ with representatives of the LAB, *Lactobacillus helveticus* M92 and *Enterococcus faecium* L3, as well as with *S.* Typhimurium FP1 and *E. coli* 3014 (Table 3).

**Table 3:** Coaggregation ability of *Lacticaseibacillus casei* 431^®^ strains grown from lyophilised culture and an isolate from the food for special medical purposes (FSMPs) after 5 h of incubation at room temperature with lactic acid bacteria and potential pathogens as test microorganisms

Test microorganism	Coaggregation/%	
*L. casei* 431^®^	*L. casei* 431^®^ FSMPs
*Lactobacillus helveticus* M92	(43.2±0.6)^b^	(57.7±1.1)^a^
*Enterococcus faecium* L3	(35.1±0.8)^b^	(44.8±0.9)^a^
*Salmonella* Typhimurium FP1	(18.4±0.9)^b^	(25.7±1.3)^a^
*Escherichia coli* 3014	(23.7±1.1)^b^	(37.2±0.7)^a^

The results are expressed as the mean value±standard deviation of three separate experiments. Different letters in superscript indicate statistically significant differences among data in the same column (Tukey’s HSD test, p<0.05, *N*=3)

The coaggregation with related LAB was higher than with the test microorganisms, where the coaggregation with *L. helveticus* M92 was approx. 10 % higher than with *E. faecium* L3 (Table 3). In all coaggregation pairs, the coaggregation percentage was higher with *L. casei* 431^®^ isolated from the FSMP matrix, which indicates the influence of the food matrix on the ability of coaggregation. Autoaggregation and coaggregation prevent colonization of pathogens on surfaces, because these processes are involved in the second phase of biofilm formation. The ability to form multicellular aggregates has been observed in numerous bacterial species, including lactobacilli. Autoaggregation and coaggregation are very important phenomena in several ecological niches. Autoaggregation of lactobacilli is necessary for their adhesion to epithelial cells and mucosal surfaces and it is a desirable property of probiotic bacteria. In the same way, the coaggregation between lactobacilli and pathogenic microorganisms contributes to the formation of a barrier that prevents pathogen adhesion to the epithelium and subsequent access to tissues, thus creating an important host defense mechanism against infections in the urogenital and GIT (*46*). Numerous studies have confirmed the beneficial effect of probiotic strains in biofilm formation, including increased resistance to temperature or low pH value (*47*). Gómez *et al.* (*48*) confirmed the potential of probiotic LAB to control the formation of *L. monocytogenes*, *S.* Typhimurium and *E. coli* O157 biofilms. Falagas and Makris (*49*) proposed the use of probiotic LAB strains for the creation of protective biofilms and their implementation in daily cleaning products to reduce the occurrence of pathogenic microorganisms.

**Fig. 4:**
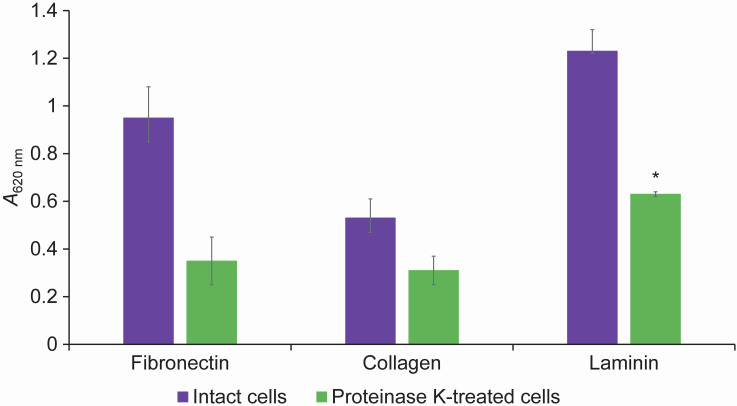
Effects of proteinase K treatment on the binding of *Lacticaseibacillus casei* 431^®^ cells to immobilised extracellular matrix proteins: fibronectin, collagen and laminin. The results are the mean value of three independent experiments and error bars represent standard deviations. *Data comparisons (intact cells *versus* proteinase K-treated cells) considered statistically significantly different (p<0.05)

The successful survival of probiotic strains in the large intestine, where they exert a beneficial effect on the host's health, depends on their adhesion to epithelial cells, cells of the intestinal and gastric mucosa, and to the extracellular matrix (ECM), which consists of various proteins, such as laminin, fibronectin and collagen. Probiotic bacteria express adhesins on the cell surface, which mediate their adhesion to components of the ECM. Pathogens also interact with ECM proteins *via* surface-exposed adhesins, which limits peristalsis and enables tissue invasion and infection. An example is a *Staphylococcus aureus* collagen-binding protein Can, known as a major virulence factor (*50*). Probiotic bacteria capable of adhering to the ECM can therefore outcompete pathogens in adherance to the surface of the host and thus prevent their penetration into the tissue. Next, the potential of *L. casei* 431^®^ to adhere to human collagen, fibronectin and laminin was investigated (Fig. 4).

The abillity of *L. casei* 431^®^ to adhere to specific ECM proteins decreased in all cases after proteinase K treatment. This indicates that the mediators of adhesion to the ECM are of a proteinaceous nature. Among the three ECM proteins, the highest cell adhesion was recorded for laminin. One can hypothesise that specific protein structures of *L. casei* 431^®^ are involved. The host-probiotic cell interaction mediated by surface proteins is the basis for the creation of a barrier that inhibits pathogen adhesion to the intestinal mucosa, thereby preventing pathogen colonization, which ultimately affects pathogen elimination from the intestinal environment. By sharing common host receptors, *Lactobacillus* strains could enable the exclusion of pathogens from the gastrointestinal or urogenital tract by direct competition for binding sites of epithelial cell proteins (*51*). In this context, the autoaggregation-promoting protein AggLb, located on the surface of bacterial cells, is involved in the interaction of *Lacticaseibacillus paracasei* ssp. *paracasei* with collagen and fibronectin (*44*, *52*). Deletion of the *aggLb* gene leads to loss of the ability to form cell aggregates, while overexpression increases autoaggregation, hydrophobicity and collagen binding potential. PCR screening with sets of primers constructed on the basis of the *aggLb* gene of *Lacticaseibacillus paracasei* ssp. *paracasei* BGNJ1-64 enabled the detection of the same type of *aggLb* gene in five different *Lactobacillus* strains that have the ability to aggregate. Heterologous expression of the *aggLb* gene confirmed the key role of the AggLb protein in cell aggregation and specific binding to collagen, which indicates that AggLb can mediate effective host colonization and prevention of pathogen colonization (*44*).

**Fig. 5:**
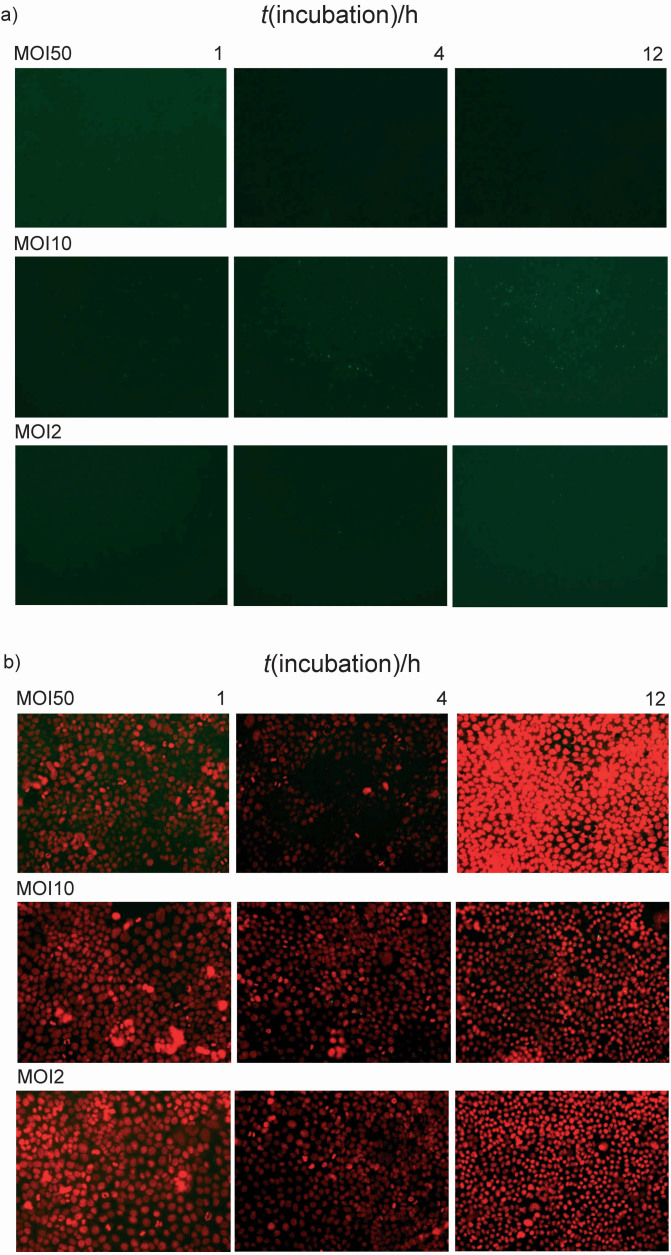
Fluorescence microscopy of adherent *Lacticaseibacillus casei* 431^®^ cells to the Caco-2 monolayer: a) only bacterial cells *L. casei* 431^®^ (green fluorescence) and b) double fluorescent layers of Caco-2 cell nuclei (red fluorescence) and bacterial cells *L. casei* 431^®^ (green fluorescence) are shown for different inoculation densities (MOI 2, MOI 10, and MOI 50) after 1, 4 and 12 h of incubation at 20× magnification. MOI=multiplicity of infection

One of the principles for selecting a potential probiotic strain is its ability to adhere to the GIT mucosa of the host. Adhesion is a multi-step process that involves the mutual contact of the bacterial cell membrane with other surfaces and is initially based on non-specific physical interactions between these two surfaces. Adhesion to the intestinal mucosa prevents the washout of probiotic cells and enables temporary colonization, immunomodulation and competitive exclusion of pathogens. The ability to adhere to the intestinal epithelium is mainly investigated in *in vitro* experiments with human intestinal cultures of cell lines (*53*). After evaluating the autoaggregation potential and the ability to bind to ECM proteins, the ability of *L. casei* 431^®^ to competitively exclude *S. enterica* serovar Typhimurium FP1 and *E. coli* 3014 was investigated using Caco-2 cell line. *In vitro* adhesion was tested using fluorescent staining of living cells adhered to the Caco-2 cell line monolayer. Adhesion was tested at MOI 2, 10 and 50 during 1, 4 and 12 h of incubation (Fig. 5).

The results show that the number of adherent cells increased with the longer incubation time, with optimal results obtained at MOI 10. Previous studies have shown that *Lacticaseibacillus paracasei* strains adhere to the Caco-2 cell line (*39*, *54*). *L. paracasei* ssp. *paracasei* KNI9 inhibited the adhesion of *Y. enterocolitica* ssp. *enterocolitica* to Caco-2 cells. AggLb of *L. paracasei* ssp. *paracasei* is not only involved in the interaction with ECM components but also in the competitive exclusion of pathogens by *L. paracasei* ssp. *paracasei* strains (*44*). In addition, the exopolysaccharide produced by *Lacticaseibacillus paracasei* ssp. *paracasei* strain has also been shown to be involved in adhesion to intestinal epithelial cells, thereby reducing the adhesion of *E. coli* to Caco-2 cells (*40*).

When testing the ability of competitive exclusion of pathogens with the probiotic strain *L. casei* 431^®^, the results showed that the adhesion of *L. casei* 431^®^ to the Caco-2 cell line reduced the attachment of *E. coli* 3014 by 1.81 log CFU/mL and the attachment of *S.* Typhimurium FP1 cells by 1.85 log CFU/mL (p<0.05), which is consistent with the results of Jankowska *et al.* (*41*), where the strain *Lacticaseibacillus paracesei* IBB2588 inhibited the binding of *Salmonella enterica* to the Caco-2 cell line. Further research of *L. casei* 431^®^ can also focus on the anticancer effect as an additional probiotic property obtained by *L. paracasei* K5, a strain with adhesion ability but also with antiproliferative and apoptotic activity on Caco-2 colon cancer cells *via* modulation of the expression of specific proteins of the Bcl-2 family (*55*).

## CONCLUSIONS

Sequencing of the 16S rRNA gene confirmed the presence of the *Lacticaseibacillus casei* 431^®^ strain in the matrix of foods for special medical purposes (FSMPs). Survival under adverse gastrointestinal tract conditions, ability to aggregate, adhesion to proteins of extracellular matrix and Caco-2 cells along with antagonistic activity indicated the competitive exclusion of *S.* Typhimurium FP1 and *E. coli* 3014 by *L. casei* 431^®^. Additionally, the FPMP matrix positively influenced cholesterol assimilation by *L. casei* 431^®^. According to the results, the incorporation of this probiotic strain in the FSMP matrix improved its probiotic activities and functional value. Therefore, by adding the probiotic strain to the innovative FSMP matrix, a synergistic effect was achieved that contributed to the improved functionality of the developed product.
